# Supplementary material for: From Planning Stage Towards FAIR Data: A Practical Metadatasheet For Biomedical Scientists
Source: Sci Data. 2024 May 22;11:524. doi: 10.1038/s41597-024-03349-2 (PMC11111677; doi:10.1038/s41597-024-03349-2)
Supplement: Supplementary file 1 — Figure S1 [file 41597_2024_3349_MOESM1_ESM.pdf]

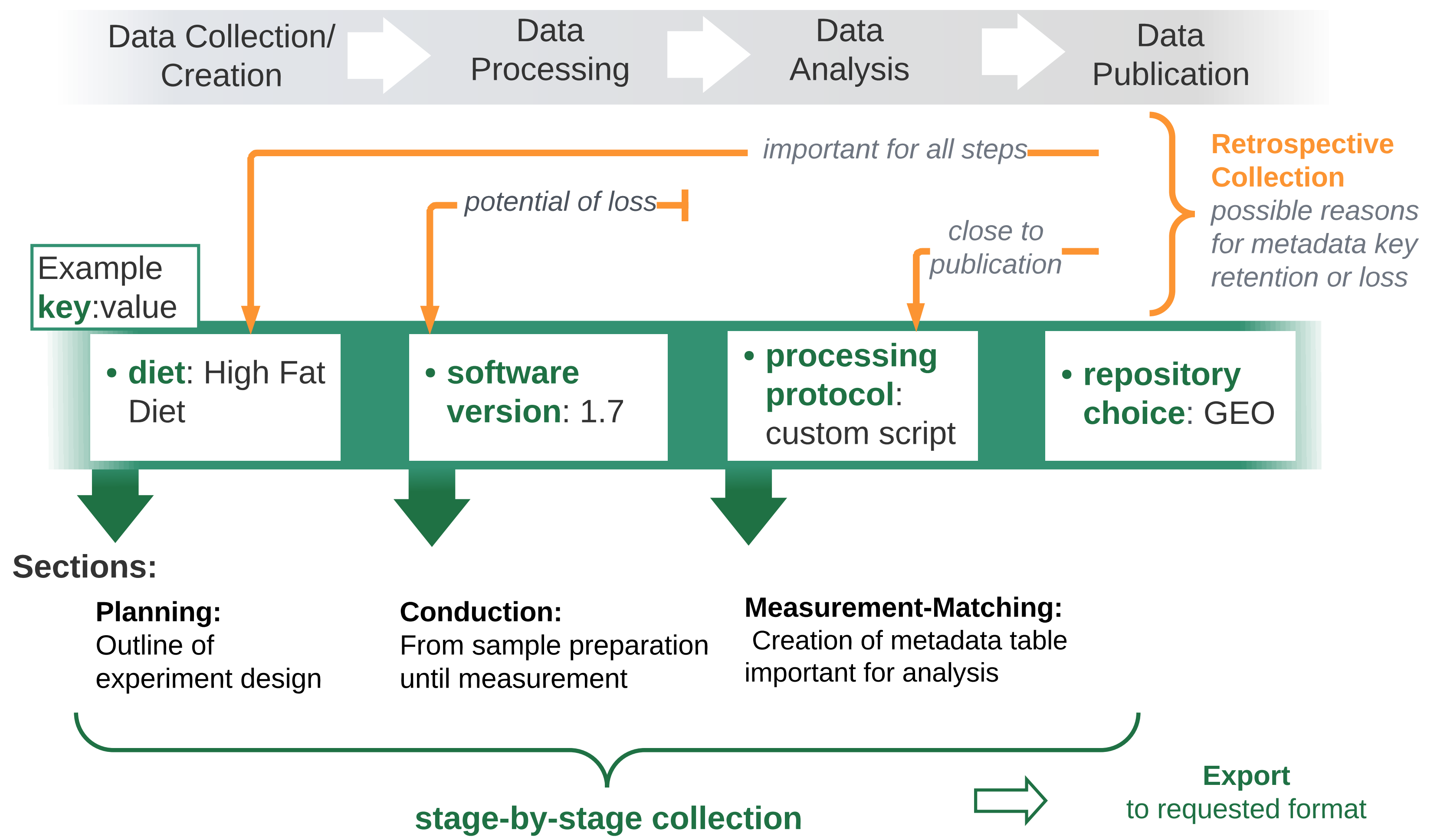

Data Collection/ Creation
Data Processing
Data Analysis
Data Publication
important for all steps
potential of loss
close to publication
Retrospective Collection
possible reasons for metadata key retention or loss
Example key:value
• diet: High Fat Diet
• software version: 1.7
• processing protocol: custom script
• repository choice: GEO
Sections:
Planning: Outline of experiment design
Conduction: From sample preparation until measurement
Measurement-Matching: Creation of metadata table important for analysis
stage-by-stage collection
Export to requested format
