# Supplementary material for: From Planning Stage Towards FAIR Data: A Practical Metadatasheet For Biomedical Scientists
Source: Sci Data. 2024 May 22;11:524. doi: 10.1038/s41597-024-03349-2 (PMC11111677; doi:10.1038/s41597-024-03349-2)
Supplement: Supplementary file 2 — Supplementary [file 41597_2024_3349_MOESM2_ESM.pdf]

1 Supplementary Material

1.1 Set of example Metadatasheets

In this section, several complete showcases are briefly described. Respective specific features highlighting a certain aspect of the proposed Metadatasheet are further discussed. You can find all complete Metadatasheets on Zenodo as well as all accompanied protocols files, if publicly available. As the Metadatasheet is to be filled alongside the data-lifecycle, majority of the measurement data as well as some protocols are under non-disclosure. Note, that the provided folder structure within each showcase is also as expected by the Metadatasheet:

Bulk RNAseq from mouse samples with a one-fold experimental design:

This example is associated with a publicly available dataset generated by Elvira Mass research group, accessible through the GEO Series accession number GSE237408. This dataset was created as part of a project aimed at understanding how maternal obesity affects the development of Kupffer cells and its implications for fatty liver disease within their offspring. The detailed example is part of a collection and linked via the value given in the 'title' key.

The data shown here involves mouse as the experimental system and bulk-RNA sequencing as the measurement technique. We compared different diets, which are part of a more complex design involving maternal diet, maternal diet during lactation, and offspring diet. Respective new terms for the controlled vocabulary were introduced. While we could have chosen a two-fold design utilizing comparison group 'others', combining maternal diet and offspring diet, we opted for keeping all diet related groups within the same comparison group to enhance the findability of the data.

Note that 'Kupffer Cells' were a constant factor in this study. There's a similar experimental design for hepatocytes in the collection. Our Metadatasheet could handle both within the same Metadatasheet by using subsamples, as indicated in figure **??** in the main manuscript. However, since this Metadatasheet was also submitted to GEO independently, we decided to keep the same setup as in the repository for consistency.

Lastly, the specified procedure used in this study is taken as an example from the controlled vocabulary. This specification is accompanied by the relevant parts of the manuscript, which are stored on a consortium-wide file location (protocol hub) under the selected value for the procedure. For completion, the protocol is added as if it would have been specified as filename. In this example, we don't have any covariates to consider, so the Measurement-Matching section contains only default rows.

Bulk metabolomics from cell-line samples with a one-fold experimental design:

This example is associated with the public available dataset generated within Karsten Hiller group. The dataset was created as part of a project aimed to analyse the glucose metabolism within human monocyte derived macrophages in response to LPS stimulation.

The data shown here involves human-derived as experimental system. The measurement technique is 13C labelling, done by the department of Bioinformatics and Biochemistry, which as no special appointed measurement-type but is associated with the type 'bulk-metabolomics' and associated dependent keys. The comparison group involves LPS treatment, at differing timepoints, which are independent of each other, hence no nesting was used.

Further, the specified protocols are provided as an additional file, the filename of which is specified in the Metadatasheet.

Bulk proteomics from mouse samples with a one-fold experimental design:

This example is associated with the data obtained from Anne-Katrin Gellner. The dataset was created as part of a project to decipher the role of stress vulnerability in the disruption of motor cortical neuroplasticity.[?].

The data shown here involves mice as an experimental system, which are exposed to a certain type of stress or not. The measurement technique is a proteomics analysis of the mice's cerebrospinal fluid (CSF). After stress-treatment, mice were classified into different phenotypes based on behavioural tests. Note, that these phenotypes are not the comparison groups, as this assignment is made on additional data and not set *a priori*. Moreover, not all samples taken from mice have associated data due to different 'reason of drop out', hence provided processed data encompasses a subset of provided mice IDs.

Bulk proteomics from human-derived samples with a one-fold experimental design:

This example is associated with unpublished data and results generated with Alexander Pfeifer's group. The dataset not yet encompassed human-derived material as experimental system, whereby the studied subjects differ in their sporting activities. Upon consultation, the on 'human-derived' keys did not encompass yet the key 'cellular component', which was needed as extracellular vesicles were studied. Leveraging on the Metadata Workbook capabilities, we added immediately the missing dependent key to the list within the 'dependent fields' sheet at the column of human-derived as experimental system. Further,

we added a set of controlled vocabulary, by appending the new introduced key 'cellular component' to the 'validation' sheet, and providing a list of plausible terms. After this, we could further fill out the Metadatasheet, having only a minor delay in recording.

Further, data were recorded 'pre' and 'post' from the same individual, yielding a nested design. Hence, the time-point of measurement defines the subsamples.

Note, within the shown example, certain variables were left out due to confidentiality as this is an unpublished study. The provided measurement data outlines the structure of the data.

16S rRNA from rat samples with a one-fold experimental design:

This example is associated with a public available 16S rRNA dataset generated within the group of Wiebke K. Fenske. The dataset was created as part of a project aimed at understanding the relationship of bariatric surgery as the most effective therapy for weight loss and type 2 diabetes remission and the role of gut microbiota in mediating the surgery's metabolic benefits[?].

The experimental system studied is 'rat' which has not been part of the experimental system collection upon consultation. As in previous showcases described, we added the missing experimental system to the list, providing requested keys and their respective controlled vocabulary, where necessary. The performed intervention was for now added to the ambiguous 'other' group, allowing free text to describe each instance. As the 'other' group might become very diverse, a group or a consortium might want to bundle a homogeneous group and create a new comparison group, analogous to the already present comparison groups, e.g., 'diet' or 'temperature'. The introduction of a new comparison group, upon ontology adjustment, will help others to more easily find related experimental set-ups among a set of Metadatasheets.

The analysis of gut microbiota composition by 16S rRNA sequencing was conducted by the company BGI (Hong Kong, China). The provided 'personal_IDs' within the Measurement-Matching section matches with the public available dataset associated with the BioProject: PRJNA735921.

Indirect calorimetry from mouse samples with a one-fold but nested experimental design:

This example is associated with a public available dataset generated within the group of Alexander Pfeifer. The dataset was created to study the energy expenditure via extracellular inosine within apoptotic brown adipose tissue[?].

The studied experimental system was 'mouse' as living animals. Hence, several further keys within the 'experimental system' segment, such as tissue type, are not applicable and marked as such. The contrasting setting was the genotype of the respective mice. For the actual measurement (indirect calorimetry) the mice were separated into single cages. This is noted within the Time-Dependence-Segment as 'interruption-type continued', as the genotype has not changed, but the animals were relocated before the actual measurement, which can result in stress. Indirect calorimetry measurements are obtained over the time course of a day, of a single mouse. Hence, time points are subsamples and present a nested design. Weight was recorded as a covariate and only measured once for each mouse and not at each time point. By design, covariates are captured at the lowest level, leading to duplicated weight measures.

Processed data is provided as Excel table, including statistical testing. Note, that the ID matching involves Sample IDs as well as Subsample IDs. This speciality is defined within the comment key of the Data File Linkage segment.

Single-cell RNA-seq from mouse with a two-fold and nested experimental design:

This example is associated with unpublished data and results generated within the group of Marc Beyer. The dataset was created to study the transcriptional programming of adipose tissue macrophages during metaflammation. There are two comparison groups, - diet, including 4 different diet regimes, and genotype, including 2 different genotypes. The Timeline-Dependence segment was used to further specify the different diet regimes, as they differed in time mice received a high fat diet. Measurements were taken on three tissues, specified using 'subsamples'. As measurement type 'scRNA-Seq' was selected, however, data is under non-disclosure. Example IDs are provided in respective fields.

Single-nucleus RNA-seq from mouse with a one-fold but nested experimental design:

This example is associated with unpublished data and results generated within the group of Tobias Fromme. The dataset was created to study the enhanced beige adipocyte detection by single nuclei RNAseq within a luciferase reporter mice. The comparison group is 'genotype'. Measurements were taken on two differing tissues, specified using 'subsamples'. As measurement type 'scRNA-Seq' was selected, as single-cell and single-nucleus measurements differ in the measured component but not with respect to the machine or other herein measurement-dependent keys. To clarify the difference, the preparation comment field and/or the measurement-dependent comment field should be used.

Provided data encompasses the raw FASTQ files for each subsample, as well as the processed data from cellranger software, which is commonly used as input for bioinformatic analysis. Note, to reduce data storage, a picture of actual raw data filenames (despite actual files) is provided. Also, a screenshot of processed data folders is provided. Those folders are named appropriate and hold multiple processed files.

Bulk lipidomics from mouse samples with a two-fold and nested experimental design:

This examples is associated with unpublished data, protocols and results generated within the group of Ludger Scheja. Here, two comparison groups are present, 'diet' and 'genotype' rendering the experimental design as two-fold. Additionally, two tissue types (two subsamples) were extracted and measured from each sample.

In this study, the weight of the mice were recorded as covariate, as specified in the covariate/constants section. Within the Metadata Workbook this covariate is automatically added to the subsamples table within the Measurement-Matching section. As the weight of the mouse and not the weight of the subsamples was recorded, the weight matches the sample match and hence is duplicated on subsample level.

Note, that data as well as protocol are not yet available to the public.

Lipolysis measurements from cell-line samples with a one-fold but nested experimental design:

This example is associated with unpublished data and generated within the group of Alexander Pfeifer. The dataset was created aimed at understanding G protein-coupled receptors (GPCR) induced lipolysis in adipocytes.

The experimental system 'cell-line' was studied, measuring the lipolysis within the supernatant of the cells. The taken cells were contrasted with respect to the comparison group 'treatment', having nine instances. Each of those instances were measured with three biological and two technical replicates. The lipolysis measurements are taken within a well plate.

The lipolysis measurement type was not part of the measurement types upon consultation, therefore added as described in previous showcases. The raw measurements are provided as a single Excel sheet, whereby the IDs refer to the respective well-location and the unique experiment identifier. The processed data is provided as Excel Workbook, including necessary calculations to get to the final lipolysis read out.

UPLC-UV measurements from cell-line samples with a one-fold but nested experimental design:

This instance is affiliated with data generated within the group of Alexander Pfeifer. The investigation focused on investigating the purines release mechanism from brown adipose tissue, within the experimental system 'mouse'. The study encompassed two distinct treatments and four 'other' groups, here involving different types of virus infections. The experimental timeline of virus treatment was detailed within the Time-Dependence segment. UPLC-UV measurements, which were not originally included in the initial set of measurements, were incorporated into the Metadata Workbook leveraging its adaptability.

Within this study, two technical replicates were acquired for each biological sample, and all samples were originally prepared on a well plate. The provided dataset includes both the raw and the processed data, including a measured standard curve for each purine. It is important to note that standard measurements are not documented in the Metadatasheet records. To exemplify the integration of well-plate design into the Metadatasheet, the provided data includes the original plate design.

FRET measurements from cell-line samples with a one-fold, timeseries experimental design:

This example is associated with unpublished data generated within the group of Alexander Pfeifer. The dataset was created to investigate real-time cAMP and PDE dynamics in murine brown adipocytes.

The experimental system 'cell-line' was studied, taking fluorescence resonance energy transfer (FRET) images. The studied cells were consecutive treated with different drugs. Hence, the comparison group 'treatment' was chosen and an instance of 'timeseries' was specified. This 'timeseries' instances were further annotated with time-dependent information within the 'Time-Dependence-Timeline'-segment.

The provided raw measurements are the FRET images which are stored within a TIFF-series, with consecutive frames (not provided within Supplementary). One could have noted each frame as subsample (nested design). However, this is not how the raw data is actually provided, potentially introducing errors through dissection. Note, only one time-series and associated, processed intensity read-out is provided.

Histology from mouse samples with a one-fold experimental design:

This example is associated with unpublished data generated within the group of Dagmar Wachten. The dataset was created to perform histology analysis of white adipose tissue from a genetically modified mice.

Here, both comparison groups received a high fat diet while their genotype differed. For the two groups, the weight was captured as covariate, whereby the diet duration was held constant. Only male mice were studied.

Mentioned protocols are handed along to the respective Metadatasheet. An example filename for a real image is also provided.

1.2 Practical Guidelines for hands-on application

When establishing the Metadatasheet and Workbook within a new environment, it's likely you want to leverage on the presented capabilities for adaptability and convenience. These capabilities offer to add upon the experimental systems analysed, the measurement types used, and additional values to keys included. Furthermore, there may arise a need to incorporate additional comparison groups or limiting to a smaller predefined set of values, such as exclusively ontology terms.
However, to fully realize their benefits, it's crucial to keep accompanying resources, like the Validation, Dependent Fields sheet and the Workbook itself, up to date and readily accessible to all users of the Metadata Workbook. Achieving FAIR data involves additional steps beyond completion of the Metadata Workbook. It necessitates the upload of both data and metadata to suitable repositories. Moreover, enhancing reproducibility relies on providing detailed protocols, which, although linked within the Metadatasheet, may not always be sufficient in terms of content. Therefore, ensuring FAIRness ultimately falls to the responsibility of individuals who provide data, metadata and associated protocols.

In the following sections, we outline our vision for utilizing the Metadatasheet and Workbook within a consortium comprising multiple groups.

Initial Set Up

Upon first use, the group should evaluate:

1. Evaluate the presence of all required experimental systems (if possible, past and future)
2. Consider extending respective dependent fields if necessary to accommodate additional requirements (e.g. if a new experimental system or measurement type is added)
3. Consider extending the predefined Comparisons groups suitable to the studied context
4. Determine and decide whether to enforce the annotation with suitable ontologies and their respective terms. This may involve creating a group-specific Validation Sheet.
5. Define which user group is authorized to add entries to the Validation and dependent fields after the initial setup and consultation. If restricted, designate responsible individuals to merge sheets and update the Workbook accordingly, ensuring accessibility for all users. This curation task involves verifying added terms for duplicates or variations and providing assistance for new keys.
6. Ensure accessibility of training material for self-learning for new users

Once the initial setup is completed, the resources are ready for distribution among group members. The setup should be suitable to the diverse experimental settings commonly encountered within the lab. Additionally, with assigned responsible individuals, scenarios such as updating resources or the Workbook itself, can be easily handled.

Distribution of resources

The Metadata Workbook should reside on a shared resource platform, such as Sciebo, granting download-only access to the normal user-group. When planning a new experiment, individuals copy the current workbook version to facilitate writing and editing. Metadatasheets are then filled out stage-by-stage and potentially shared among involved parties to complete respective sections. In the event of a missing value for a key, users are empowered to update the Validation sheet, leveraging the provided adaptability to supplement lists. However, in most cases this should not be necessary if the initial set up is done carefully.
Upon completion, the Metadatasheet can be exported as a *xlsx* sheet, to be stored alongside the data. This ensures that links within the sheet, such as protocols, remain accurate, and enables proper matching of data within the measurement-matching section. For long-term storage and security, it's advisable to avoid local disks and instead utilize shared filesystems like Sciebo. Adhering to the suggested naming convention of Metadatasheets is favourable. The naming convention allows programs to 'crawl' these filesystems, gathering all existing Metadatasheets. This gathering accompanied by our provided ontology can serve as the foundation for a comprehensive group, topic, or consortium-wide database.
Additionally, the Metadata Workbook facilitates the generation of GEO's requested metadata format. For repositories reliant on a Graphical User Interface for upload, the Metadatasheet acts as a single-point lookup, streamlining the process.

Additional steps for publishing FAIR-data

To ensure the publication of FAIR data, several additional steps lie within the user's responsibility. First, it's essential to employ ontology terms wherever possible, even if not strictly enforced by others, to enhance data findability and interoperability. Second, detailed procedure protocols facilitate reproducibility and transparency. Within the Metadata Workbook protocols are only linked but not structured themselves. Third, both raw data with its metadata should be uploaded to a suitable repository. The Metadata Workbook offers straightforward deposit procedures to platforms like GEO. We aim to extend on those deposition procedures. However, in cases where no suitable repository exists, careful consideration must be given to alternative options, such as establishing a custom topic-centered database. In certain instances, uploading to domain-unspecific repositories could serve as a solution. While this establishes the accessibility of your data, it may result in low findability.

The proposed measures, lying within the user's responsibility, collectively contribute to the findability, accessibility, interoperability, and reusability of research data.

Metadatasheet-Keys	Suitable Ontologies or formal means
Name of Mouse Strain	National Cancer Institute Thesaurus
Name of Mouse Line	National Cancer Institute Thesaurus
Name of Cell Line	Cell line ontology
Genotype of Mouse	MGI - nomenclature
Tissue Type	Brenda Tissue Ontology
Measurement Type	Experimental Factor Ontology (EFO)
Technology	Ontology for Biomedical Investigations
Cell Types	Cell Ontology
Experimental System	Ontology for Biomedical Investigations
Procedure (Standard Operation Protocols)	Experimental Factor Ontology (EFO)

Table S1. Metadatasheet-Keys and Suitable Ontologies

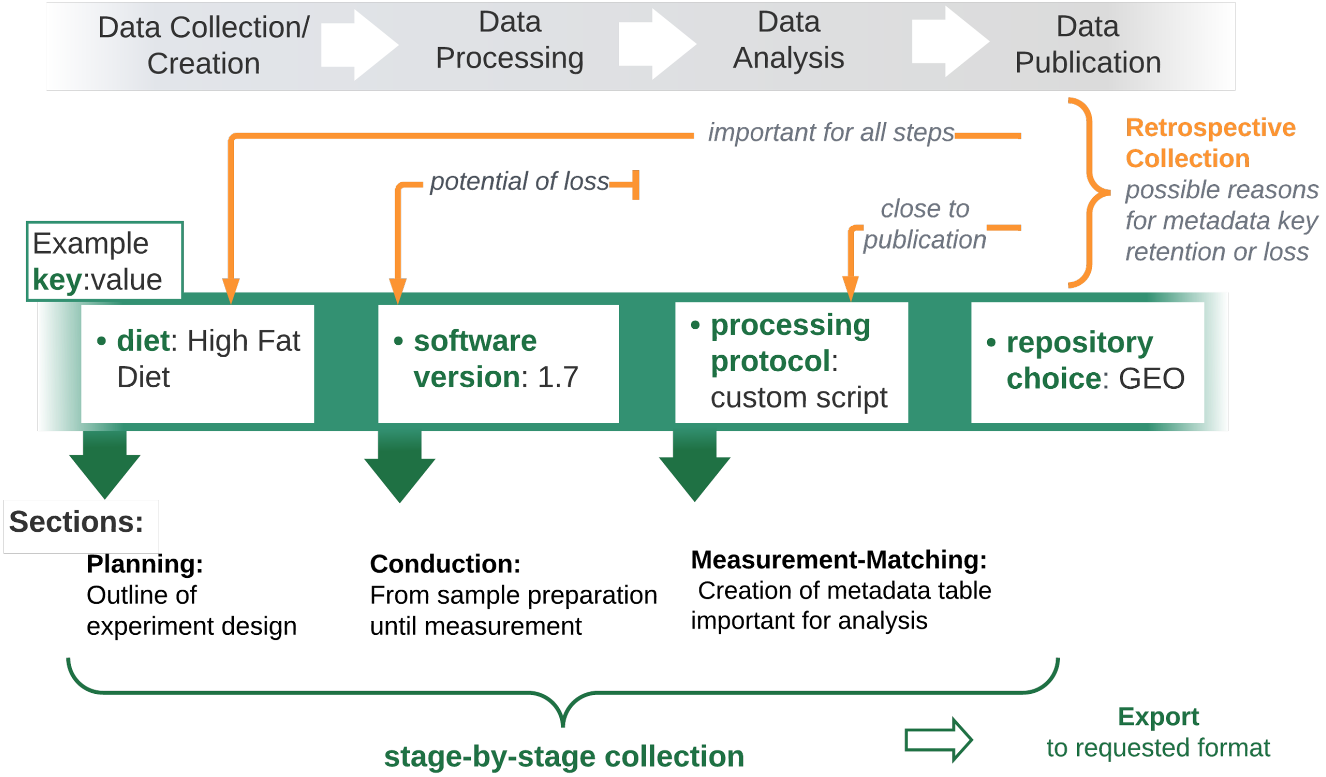

Figure S1. Challenges of retrospective collection. Through the retrospective collection, recovering necessary metadata items can be challenging. When not generally recorded, only metadata-items of immediate interest for the next step or for data analysis will be available, other items that ensure FAIRness, e.g., the software version of a specific program, might not get recorded at all and will hence be lost. A stage-by-stage approach along the Data Lifecycle is capable to resolve the challenges.
